# Joint Fiber Nonlinear Noise Estimation, OSNR Estimation and Modulation Format Identification Based on Asynchronous Complex Histograms and Deep Learning for Digital Coherent Receivers

**DOI:** 10.3390/s21020380

**Published:** 2021-01-07

**Authors:** Shuailong Yang, Liu Yang, Fengguang Luo, Bin Li, Xiaobo Wang, Yuting Du, Deming Liu

**Affiliations:** The School of Optics and Electronics Information, Huazhong University of Science and Technology, Wuhan 430074, China; slyang20201210@163.com (S.Y.); fgluo@hust.edu.cn (F.L.); libin8208@126.com (B.L.); M201872131@hust.edu.cn (X.W.); xtdythust@163.com (Y.D.); 15827468251@163.com (D.L.)

**Keywords:** optical performance monitoring, fiber nonlinear, multi-tasking artificial neural networks

## Abstract

In this paper, asynchronous complex histogram (ACH)-based multi-task artificial neural networks (MT-ANNs), are proposed to realize modulation format identification (MFI), optical signal-to-noise ratio (OSNR) estimation and fiber nonlinear (NL) noise power estimation simultaneously for coherent optical communication. Optical performance monitoring (OPM) is demonstrated with polarization mode multiplexing (PDM), 16 quadrature amplitude modulation (QAM), PDM-32QAM, as well as PDM-star 16QAM (S-16QAM) for the first time. The range of launched power is −3 to −2 dBm with a fiber link of 160–1600 km. Then, the accuracy of MFI reaches 100%. The average root mean square error (RMSE) of OSNR estimation can reach 0.37 dB. The average RMSE of NL noise power estimation can reach 0.25 dB. The results show that the monitoring scheme is robust to the increase of fiber length, and the solution can monitor more optical network parameters with better performance and fewer training data, simultaneously. The proposed ACH MT-ANN has certain reference significance for the future long-haul coherent OPM system.

## 1. Introduction

With the development of optical fiber communications, optical performance monitoring (OPM) is essential to ensure high-quality services and reliable optical networking for long-distance coherent optical systems [1,2,3]. As transmission distance and transmission power increase, distortion caused by fiber nonlinearity (NL) noise and gaussian random white noise are the main limiting factors for optical transmission and cannot be ignored in long-distance larger-capacity optical networks [4,5,6,7]. Therefore, it is essential to realize optical signal-to-noise ratio (OSNR) estimation and nonlinear noise power estimation in order to accurately ensure the quality of optical signals and transmission links for future long-distance larger-capacity flexible and transparent optical communications in an elastic optical network [8,9,10].

In recent years, deep learning (DL) technologies that include a convolutional neural network (CNN), deep neural network (DNN), and recurrent neural network (RNN) have been widely used in OPM due to their self-learning ability [11,12]. The OPM based on DL can be divided into two aspects: single task (ST) and multi-task (MT) OPM. (1) ST OPM: The artificial neural networks (ANN) and DNN scheme based on asynchronous histograms (AHs) have been proposed for estimating OSNR in an optical network [13,14]. In order to estimate the NL noise power characteristic of a long-distance optical network, several NL noise-power monitoring methods using a high-speed, coherent receiver based on ST machine learning (ML) and ST long short-term memory (LSTM) have been reported in simulation results [15,16,17,18]. (2) MT OPM: MT-CNN extracts features from eye diagrams (EDs) to achieve an OSNR estimation and MFI [19,20]. As the OSNR decreases, EDs become less obvious. The MT-ANNs, combined with amplitude histograms, were proposed to obtain a better feature database compared to EDs, which have high complexity and limited features [21]. A monitoring scheme based on Stokes axes combined with MT-DNN was proposed to realize the OSNR estimation and MFI [22]. A simple MT-DNN OPM with AHs was used for a joint OSNR estimation and MFI from a directly detected polarization mode multiplexing (PDM) and higher modulation format (64-QAM) signals to meet more optical network systems and simplify the construction cost [23].

At the same time, a simple and efficient joint OSNR and NL estimation scheme based on training sequence was proposed at the expense of communication capacity [24]. Some low-complexity MFI or OSNR estimation schemes characterized by the cumulative distribution function of the received signal amplitude have also been proposed [25,26,27,28]. In our previous research work, MT-DNN was proposed for joint OSNR estimation, MFI and bit rate identification (BRI) to enrich the OPM parameters [29,30]. In summary, these methods more or less have the following disadvantages: (1) To our knowledge, these proposed MT-DL-based OPM methods are mainly focused on the OSNR estimation for short-distance or back-to-back transmission without considering the effects of fiber NL noise. (2) The existing NL noise-power estimation technologies are based on a single-task ML and very complex LSTM. ST OPM is difficult to meet the requirements of multiple parameters monitoring, and space of LSTM grows exponentially with symbol sequence length [31]. (3) NL noise power estimation is only proposed and demonstrated by ST-DL and large sampling data. (4) The previous OPMs are focused on the QAM modulation format, without considering other modulation formats, such as the star-QAM, which is usually utilized in long-haul optical packet switching communications. Therefore, an efficient simultaneous NL noise power and OSNR estimation scheme based on the DL network is needed for future optical communication systems.

In our paper, asynchronous complex histograms (ACHs) with MT-ANN were designed to realize NL noise power estimation, OSNR estimation and MFI for the higher modulation format optical network link simultaneously. First, the ACHs containing richer channel impairment information were obtained with low-speed analog-to-digital converters (ADCs). Then, the MT-ANN, based on ACHs, was carried out on a long-distance coherent optical fiber transmission system with few training data and low-speed ADCs. The launched optical power range of each channel was −3.0 to +2.0 dBm, and the range of transmission distance was 160–1600 km. The MFI accuracy of PDM-S-16QAM, PDM-16QAM and PDM-32QAM could reach 100%. The average RMSE for the OSNR estimation was 0.37 dB. The average RMSE of the NL estimation was 0.25 dB. Therefore, this designed ACH-MT-ANN scheme provides a reference for MT OPM for future long-distance optical communications.

The remainder of this paper is structured as follows. The proposed ACH theoretical model and the ACH-based MT-ANN structure model are derived in Section 2. Section 3 introduces the system simulation settings and results. Finally, Section 4 summarizes this paper.

## 2. Operation Principle

### 2.1. Asynchronous Complex Histograms

**Figure 1:**
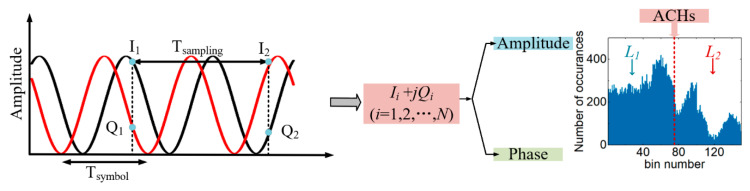
ACHs sampling principle of the PDM-S-16QAM optical network.

**Figure 2:**
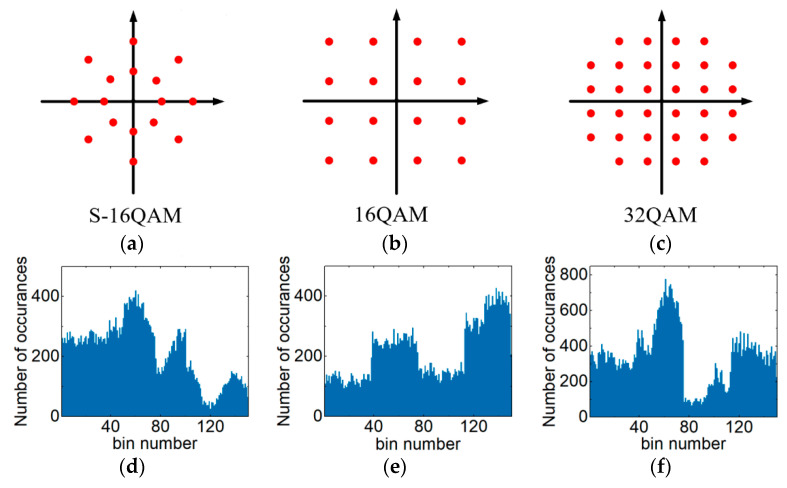
(**a**–**c**) Constellation diagram of different modulation formats: (**a**) S-16QAM, (**b**) 16QAM and (**c**) 32QAM. (**d**–**f**) ACHs with different modulation formats: (**d**) PDM-S-16QAM, (**e**) PDM-16QAM and (**f**) PDM-32QAM.

The principle of ACHs is shown in Figure 1. ACHs are statistical histograms of complex data after sampling. At the receiving end of the coherent system, the detected signal is divided into *I*/*Q* signals. The data are sampled periodically, and the sampling period is T_sampling_. One symbol period is T_symbol_. There is no relationship between the sampling period T_sampling_ and the symbol period T_symbol_. The length of the sampled data is *N*. *I_i_* and *Q_i_* at the same sampling time are combined together, as shown in Equation (1).

(1) $${Data}_{ACH}=I_{i}+jQ_{i}(i=1,2,\ldots ,N)$$

where *j* represents the imaginary part of a complex number. *Data_ACH_* represents the plural database of an ACH. The two collected signals *I_i_* + *jQ_i_* are combined according to the real and imaginary parts of the complex numbers to form the complex axis information of the ACHs. Then, the collected complex information is statistically classified to obtain the statistical histogram shown in Figure 1, where the upper horizontal axis is the plural axis information of *I_i_* + *jQ_i_*. The bottom horizontal axis is the bin number of *I_i_* + *jQ_i_*, which is the statistical point of each complex number information. The vertical axis is the number of occurrences; that is, the distribution of each complex number. The AHs contain only the amplitude information of the data [14,15]. Taking PDM-S-16QAM as an example, the complex signal contains two amplitude values [32]. The amplitude of PDM-S-16QAM divides the complex data in the ACHs graph into two parts, *L*_1_ and *L*_2_, as shown in Figure 1. Then, the phase information of *L*_1_ and *L*_2_ are respectively counted in the phase database of the corresponding area. That is, the phase information in the range of amplitudes *L*_1_ and *L*_2_ is all counted to form ACHs. Thus, ACHs contain more transmission impairment information (amplitude and phase information) compared to traditional asynchronous histograms. Figure 2a–c are constellation diagrams of different modulation formats (PDM-S-16QAM, PDM-16QAM and PDM-32QAM). Figure 2d–f show ACHs with different modulation formats (PDM-S-16QAM, PDM-16QAM and PDM-32QAM). Figure 2a show the constellation of PDM-S-16QAM. Figure 2d show the ACH of the PDM-S-16QAM signal through the transmission link. For different modulation formats, the amplitude and phase of the signal are different. Therefore, the plural axes of the ACHs are not consistent.

**Figure 3:**
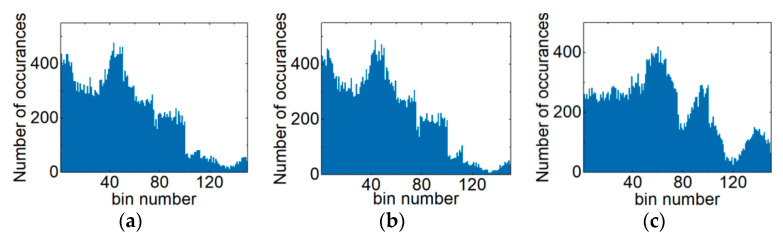
ACHs with different OSNRs in the PDM-S-16QAM system: (**a**) OSNR value is 21 dB, (**b**) OSNR value is 25 dB and (**c**) OSNR value is 30 dB.

**Figure 4:**
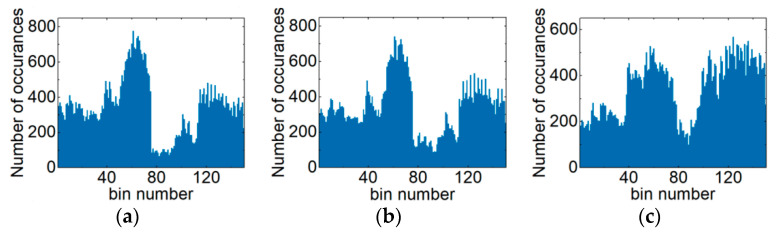
Collected ACHs of the PDM-32QAM system: (**a**) the optical fiber input power is −3 dBm, (**b**) the optical fiber input power is −1 dBm and (**c**) the optical fiber input power is 2 dBm.

Figure 3 is diagram of the ACHs in the PDM-S-16QAM system. The respective OSNRs of Figure 3a–c are 21 dB, 25 dB and 30 dB. For the 1000 km PDM-32QAM optical network transmission link, the ACHs are shown under different optical fiber input powers. Figure 4a–c are ACHs with −3 dBm, −1 dBm and 2 dBm input fiber power, respectively. As can be clearly seen from Figure 2, Figure 3 and Figure 4, ACHs depend on the modulation format, OSNR and NL. Therefore, ACHs can be used to monitor MFI, OSNR and NL.

### 2.2. Asynchronous Complex Histogram MT-ANN

**Figure 5:**
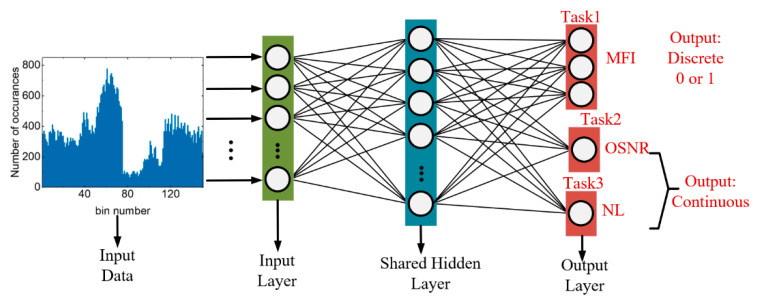
Schematic diagram of MT-ANN.

With the help of multi-task deep learning algorithms, ACH MT-ANNs can achieve three tasks simultaneously. As shown in Figure 5, the input data are the collected ACHs, which are 150 × 1 arrays. The number of neurons in the input layer and shared layer is 150 and 200, respectively. The hidden shared layer of MT-DNN uses sigmoid to activate neurons. In the output layer, continuous output tasks (OSNR estimation and NL noise power estimation) use direct output functions. The classification output task (MFI) uses the Softmax function. Different activation functions and neurons are used to accomplish different tasks.

OPM includes three tasks, namely MFI for three modulation formats identification, OSNR and NL noise power estimation. The loss function *L* of MT-ANN is shown in Equation (2). Among them, *L*_1_, *L*_2_ and *L*_3_ are the loss functions of OSNR estimation, MFI and NL noise power estimation, respectively. λ_1_, λ_2_ and λ_3_ are the weights of these three tasks, respectively. Then, Equations (3) and (4) are expressions of *L*_1_, *L*_2_ and *L*_3_ functions.

(2) $$L=\lambda_{1}L_{1}+\lambda_{2}L_{2}+\lambda_{3}L_{3}$$

(3) $$L_{2}=-\frac{1}{m}\left[\sum_{i=1}^{m}y_{i}\log{\hat{y}}_{i}+(1-y_{i})\log\left(1-{\hat{y}}_{i}\right)\right]$$

(4) $$L_{1}=L_{3}=\frac{1}{m}{\sum_{i=1}^{m}\left(y_{i}-{\hat{y}}_{i}\right)}^{2}$$

where $y_{i}$ is the actual output, and ${\hat{y}}_{i}$ is the predicted output of MT-ANN. *L*_2_ is the cross-entropy loss function, and *L*_1_ and *L*_3_ are the mean square error (MSE) functions [23].

## 3. System Setup and Results

### 3.1. System Setup

**Figure 6:**
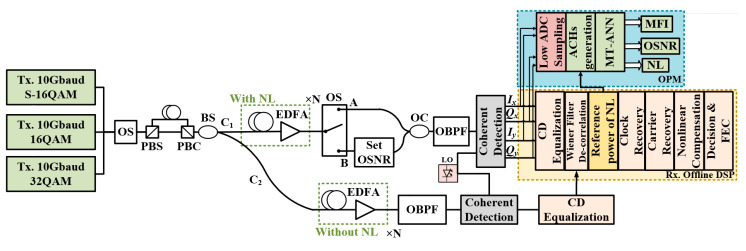
Block diagram of the OPM (MFI, OSNR and NL) based on MT-ANN for an optical fiber transmission system. (PBS: polarization beam splitter, PBC: polarization beam coupler, BS: beam splitter, EDFA: erbium-doped optical fiber amplifier, OS: optical switching, OC: optical coupler, OBPF: optical bandpass filter, LO: local oscillator, ADC: analog to digital converter).

The commercial software Virtual Photonics Inc. (VPI) Transmission Maker was used to build the long-distance, multi-channel coherent optical transmission system. The length of the sequence used was 2^15^ − 1. Then, 3 widely used optical signals (PDM-S-16QAM/PDM-16QAM/PDM-32QAM) were digitally generated, respectively. An optical switch was used to select the transmission signal of the optical link. The bandwidth of the transmission system was 10 Gbaud. Figure 6 is a block diagram of the OPM (MFI, OSNR estimation and NL noise power estimation) based on the ACH-MT-ANN for a long-distance, optical-fiber transmission system, in which the range of the transmitted power of each channel is −3.0 to +2.0 dBm. The modulated optical signal was sent to the fiber link, and each span included 80 km of standard single-mode fiber (SSMF) and an erbium-doped optical fiber amplifier (EDFA). The parameters of the system are shown in Table 1. The reference OSNR of PDM-S-16QAM, PDM-16QAM and PDM-32QAM varied within the range of 21–28 dB in steps of 1.0 dB. The total length of the transmission fiber varied from 160 km to 1600 km. At the receiver, an optical bandpass filter (OBPF) with 50 GHz bandwidth was used to filter the optical signal, which was then detected by the coherent receiver. The ACHs were obtained by low-speed ADCs sampling. Then, the ACHs were input into the ACH MT-ANNs. The OPM parameters can be input into the data recovery module, which has a reference value for data recovery. In our design, the ACH MT-ANN was built on the MATLAB platform.

In terms of the measurement of NL power, the optical signal was divided into two C_1_ and C_2_ optical signals by the BS. After C_1_ passed through the optical switch, the optical signals were marked as A and B signals. A and C_2_ are signals with and without non-linearity, respectively. At the receiving end, the coherent receiver obtained the digital signals of A and C_2,_ respectively. After the dispersion equalization, the signal containing the dispersion and the signal without the dispersion were obtained, respectively. Then, the NL noise power of the measured channel was obtained by Wiener filter decorrelation [33], as shown in Equation (5). Different nonlinear powers were obtained by adjusting the transmission distance and transmission power.

(5) $${\left|H_{r}\left(k,n\right)\right|}_{2}=\max\left(\frac{{\left|X\left(k,n\right)\right|}^{2}-\gamma {\left|\hat{N}\left(k,n\right)\right|}^{2}}{{\left|X\left(k,n\right)\right|}^{2}},\beta \right)$$

where ${\left|X\left(k,n\right)\right|}^{2}$ and ${\left|\hat{N}\left(k,n\right)\right|}^{2}$ are the power spectral estimates of noisy speech and additive noise signals, respectively (*k* and *n* are the time and frequency indices), *γ* is the noise overestimation factor and *β* is the spectral floor parameter. In order to train MT-ANN, 20,000 sets of data were collected for each modulation format, corresponding to different OSNR, linewidth, launched power and span. The entire collection set was randomly divided into a training data set (70%) and a test data set (30%).

### 3.2. Performance Discussion of OPM Based on Different ACHs Parameters

**Figure 7:**
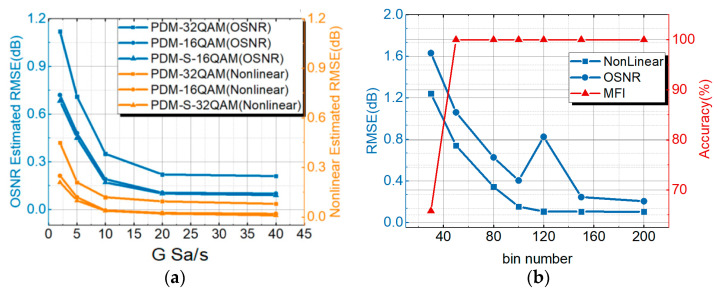
(**a**) RMSE of OSNR estimation and NL noise estimation with respect to the electronic sampling rate during the testing process for various modulation formats. (**b**) Trend of monitoring results (OSNR estimation, MFI and NL noise estimation) with the bin number of ACHs for PDM-32QAM.

As shown in Figure 7a, when the sampling rate was increased from 2 GSa/s to 40 GSa/s, the RMSE for NL noise estimation of PDM-32QAM was reduced from 0.58 dB to 0.2 dB. At the same time, for PDM-16QAM and PDM-S-16QAM, the RMSE was reduced from 0.28 dB to 0.1 dB and from 0.25 dB to 0.1 dB. The RMSE for the OSNR of PDM-32QAM was reduced from 1.18 dB to 0.27 dB. At the same time, for PDM-16QAM and PDM-S-16QAM, the RMSE was reduced from 0.78 dB to 0.17 dB and from 0.75 dB to 0.16 dB. When the electronic sampling rate increased, the inherent fluctuations of the ACHs decreased accordingly, resulting in a decrease in NL noise estimation and OSNR estimation monitoring errors. The results show that the NL noise RMSE could also reach 0.5 dB, and the OSNR was 1.1 dB when the sampling rate was reduced to one fourth (10 Gsa/s) of the signal bandwidth. In order to study the impact of the ACHs with a different data bin number on OPM results, the data bin number of the ACHs was changed to reflect changes in the OPM results. Figure 7b shows the trend of monitoring results (MFI, OSNR estimation and NL noise estimation) with the bin number of points in ACHs. The horizontal axis is the bin number of the ACHs required for training (30–200). The blue vertical axis is the RMSE results of OSNR estimation and NL noise estimation. The red vertical axis is the recognition accuracy of an MFI. As the bin number of ACHs increased, the RMSE results of NL noise power gradually decreased. When the bin number of the ACHs was greater than 80, the RMSE result of NL noise power changed little. When the bin number of ACH points was equal to 80, the monitoring results of the RMSE of the OSNR estimation appeared to be better. Considering the influence of the two parameters of epochs and ACHs on the training complexity, the bin number of the ACHs was set to 80 to obtain the monitoring performance.

**Figure 8:**
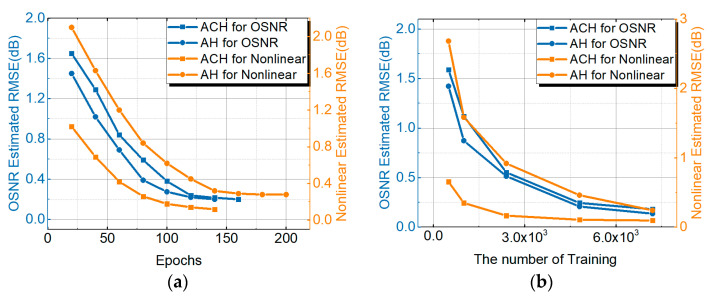
(**a**) Comparison of NL and OSNR monitoring performance between AH MT-ANN and ACH MT-ANN. (**b**) The RMSE monitoring result of OSNR and NL with the training number of AHs and ACHs for PDM-32QAM.

In previous studies, MT-ANNs combined with AHs were proposed to achieve the OSNR estimation and MFI. The NL noise estimation schemes were demonstrated based on the ST-DL with the high-speed sampling signals. The AHs were not used to estimate the NL noise. In order to demonstrate the monitoring performance of the ACH MT-ANNs, in this paper, an AH MT-ANN for NL OPM was proposed and compared with the ACH MT-ANN for PDM-32QAM. Figure 8a is a comparison of NL noise power and the OSNR estimation performance between the general AH MT-ANN and the proposed ACH MT-ANN for PDM-32QAM. Although the training efficiency of the OSNR estimation based on ACH MT-ANN was lower than that of AH MT-ANN, the OSNR estimation performance was closer to about 0.24 dB. Taking the 0.4 dB RMSE result as the standard for NL noise power estimation, the epochs for training based on the ACH monitoring network were reduced by 50% compared to the AH monitoring network. Figure 8b shows the RMSE result of OSNR and NL noise power with the number of AH and ACH training data. The RMSE standard of NL noise power and OSNR is 0.4 dB. Thus, the NL noise power estimation only requires about 1000 ACHs compared to 6000 AHs. OSNR estimation requires 2800 ACHs, and OSNR estimation requires 2400 AHs. In order to meet the optimal OSNR estimation and NL noise power estimation performance simultaneously, the maximum amount of training data for ACHs and AHs is the standard. Therefore, ACHs require 2800 training data, which is better than 6000 training data for AHs. The amount of training data was increased by 53.3%, with a 0.4 dB RMSE standard of NL noise power and an OSNR.

### 3.3. Discussion of ACH MT-ANN Parameters

**Figure 9:**
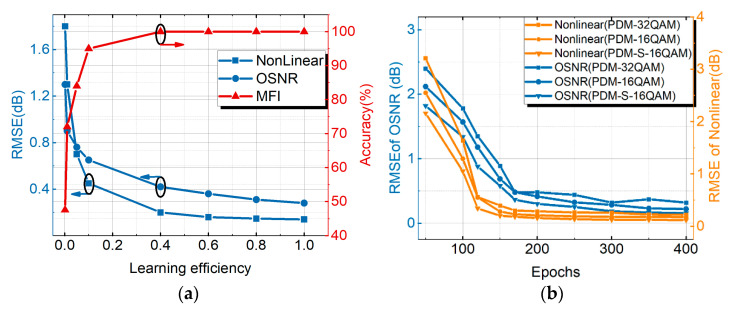
(**a**) Trend graph of learning efficiency with OPM (MFI, OSNR estimation and NL noise power estimation) results for PDM-32QAM system, (**b**) the trend of OPM with epochs for PDM-S-16QAM, PDM-16QAM and PDM-32QAM.

In the MT-ANN, learning efficiency affects the monitoring results. If the learning efficiency is changed, the effect will be reflected in the monitoring results of OSNR estimation, MFI and NL noise power estimation. As shown in Figure 9a, the horizontal axis is the learning efficiency. The blue vertical axis is the RMSE of OSNR estimation and NL noise estimation. The red vertical axis is the monitoring accuracy of MFI. When the learning efficiency was greater than 0.5, the results of OPM (OSNR estimation, MFI and NL noise power estimation) changed little. The monitoring accuracy of MFI reached 100%. The RMSE of the OSNR was about 0.4 dB. The RMSE of NL noise power was about 0.21 dB. Therefore, the learning efficiency was set to 0.8 in the MT-ANN. Figure 9b shows the trend of OPM with epochs for PDM-S-16QAM, PDM-16QAM and PDM-32QAM. When epochs were greater than 170, the RMSE of the OSNR changed little (about 0.38 dB) for the PDM-32QAM system. When epochs were greater than 110, the RMSE of NL noise power changed little (about 0.29 dB). When epochs were 170, the average RMSE of NL noise power and OSNR could reach 0.25 dB and 0.48 dB, respectively.

### 3.4. Discussion of Nonlinear Monitoring versus System Parameters

**Figure 10:**
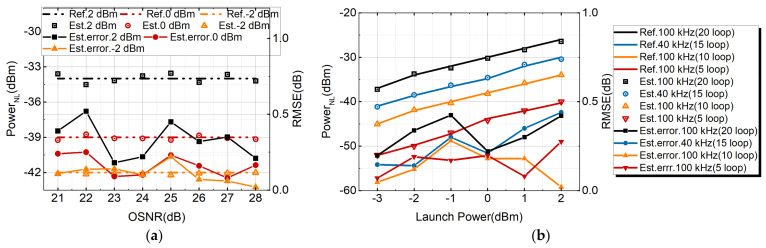
(**a**) Estimated NL noise power and error under different OSNR of 800 km PDM-32QAM. (**b**) Estimated NL noise power with different linewidth and transmission distance for 32QAM system.

Figure 10a shows the estimated NL noise power for a PDM-32QAM optical fiber communication system with an 800 km transmission distance. The laser linewidth was 100 kHz, and the launched power was −2 dBm, 0 dBm and 2 dBm. When the OSNR ranged from 21 to 28 dB, the absolute errors tested were less than 0.6 dB in the PDM-32QAM system. For different OSNRs, the estimation error was almost unchanged. Then, in order to test the stability and reliability of NL noise power estimation, the tolerance of NL noise power estimation to linewidth and the transmission length were studied. The linewidth of the laser (40 kHz and 100 kHz) and the transmission length (from 400 km to 1600 km) were changed. It can be seen from Figure 10b that the increase of 1.0 dB in transmitted power will result in an increase of NL noise power of about 3.0 dB. The power is proportional to the cubic power of the transmitted power [34]. In the PDM-32QAM system, the transmission length and laser linewidth were 5 span (400 km) and 100 kHz, 10 span (800 km) and 100 kHz, 15 span (1200 km) and 40 kHz, 20 span (1600 km) and 100 kHz. The tested RMSE was 0.24 dB, 0.28 dB, 0.36 dB, 0.23 dB. The RMSE of each training was not exactly the same, but in our method the error was within 1.0 dB.

### 3.5. Results and Discussion of MFI, OSNR and NL Noise Distortion Estimation

**Figure 11:**
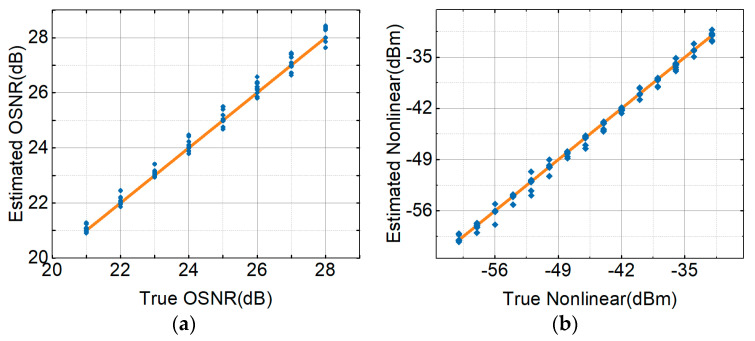
Monitoring error for all signals, (**a**) OSNR estimation, (**b**) NL noise power estimation.

The three modulations (PDM-S-16QAM, PDM-16QAM and PDM-32QAM) were selected as a reference to illustrate the performance of ACH MT-ANNs. The three mixed signals were sent into the MT-ANN to estimate the monitoring performance. Table 2 shows the recognition accuracy of the three modulation formats in the case of epochs greater than 300. The monitoring accuracy of MFI could reach 100%. Figure 11 shows the OSNR estimation, NL noise power estimation and MFI results for PDM-S-16QAM, PDM-16QAM and PDM-32QAM in the ACH MT-ANN. Figure 11a shows the OSNR estimation for all signals. The horizontal axis is the true OSNR. The vertical axis is the OSNR estimated by RMSE. The monitoring interval of OSNR was 1 dB. The monitoring range of OSNR was 21–28 dB. The RMSE of the OSNR was counted for all signals. The average RMSE of the OSNR was 0.37 dB for the PDM-S-16QAM, PDM-16QAM and PDM-32QAM signals. The RMSE of the OSNR was still within 1 dB. Figure 11b shows the RMSE of the NL noise power for PDM-S-16QAM, PDM-16QAM and PDM-32QAM in the ACH MT-ANN. The horizontal axis is the actual NL noise power. The vertical axis is the NL noise power by RMSE. The estimation range of NL noise power was −60 dBm to −32 dBm. The RMSE of NL was counted. The RMSEs of the NL noise power were 0.25 dB for the PDM-S-16QAM, PDM-16QAM and PDM-32QAM signals.

## 4. Conclusions

In this paper, we propose an OPM (MFI, OSNR estimation and NL noise power estimation) scheme based on the ACH MT-ANN for three commonly used modulation signals (PDM-S-16QAM, PDM-16QAM and PDM-32QAM). ACHs contain richer channel impairment information (amplitude and phase impairment information) compared to the AHs and can be obtained with low-speed ADCs. In the demonstration system, we conduct research from the data bin number of ACHs, the learning efficiency of MT-ANN and epochs. The performance of the ACH MT-ANN is optimized. The monitoring accuracy of MFI is 100%. At the same time, the OSNR estimation range is 21–28 dB for all signals. The average RMSE of the OSNR can reach 0.37 dB. By adjusting the transmission power of the optical network and the length of the fiber link, the NL noise power is measured. The estimation range of NL noise power is −60 to −32 dBm. The average RMSE of NL noise power can reach 0.25 dB for all signals. At the same time, the effect of the OSNR is studied on NL noise power estimation. The average RMSE of NL noise power is less than 0.6 dB. It is accurate to compare the estimated NL noise power with the reference NL noise power. For a different OSNR, the estimation error is almost unchanged. In the ACH MT-ANN, the results of NL noise power monitoring are compared under different linewidths to study the effect of laser linewidth on NL noise power estimation. The average RMSE of NL noise power is less than 0.4 dB. Due to its low cost and easy implementation, the proposed OPM solution has the potential for usage in next-generation larger-capacity coherent optical-fiber transmission systems in the elastic optical network.

## Figures and Tables

**Table 1:** The key parameters of the system.

**Modulation Format:**		16QAM, S-16QAM and 32QAM
**Signal Bandwidth:**		10 Gbaud
**Sampling Rate:**		40 GSa/s
**SSMF**	Length:	80 km
	Loop:	2–20
	Attenuation:	0.2 × 10^−3^ dB/m
	Dispersion:	16 × 10^−6^ s/m^2^
	PMD (Polarization mode dispersion) Coefficient:	0.1 × 10^−12^/31.62 s/(m^1/2^)
	Nonlinear Index:	2.6 × 10^−20^ m^2^/W
**Waveguide:**		1550 nm
**Laser Linewidth:**		1 × 10^5^ Hz

**Table 2:** The MFI recognition accuracy of the three modulation formats (PDM-S-16QAM, PDM-16QAM and PDM-32QAM).

		Identified Modulation Format		
		PDM-S-16QAM	PDM-16QAM	PDM-32QAM
**Actual Modulation Format**	PDM-S-16QAM	1908 (100%)		
	PDM-16QAM		1928 (100%)	
	PDM-32QAM			2164 (100%)

## Data Availability

Data sharing is not applicable to this article.
